# Sexual Health Education at Home: Attitude and Practice of Iranian Parents

**Published:** 2018-01

**Authors:** Jila GANJI, Mohammad Hassan EMAMIAN, Raziyeh MAASOUMI, Afsanah KERAMAT, Effat MERGHATI KHOEI

**Affiliations:** 1. Student Research Committee, School of Nursing and Midwifery, Shahroud University of Medical Sciences, Shahroud, Iran; 2. Center for Health Related Social and Behavioral Sciences Research, Shahroud University of Medical Sciences, Shahroud, Iran; 3. Dept. of Reproductive Health, School of Nursing and Midwifery, Tehran University of Medical Sciences, Tehran, Iran; 4. Reproductive Studies and Women’s Health Research Center, Shahroud University of Medical Sciences, Shahroud, Iran; 5. The Iranian National Center for Addiction Studies, Family & Sexual Health Division, Brian & Spinal Cord Injury Research Center, Institution of Neuroscience, Tehran University of Medical Sciences, Tehran, Iran

## Dear Editor-in-Chief

Sexual behaviors are common among children. Due to absence of a school and home-based sexual health education, Iranian mothers and fathers mostly, are not well-educated and trained about children sexuality (1, 2). Parents’ insufficient knowledge and incompetency to manage sexual behaviors properly would cause inappropriate reaction, which can manipulate child’s natural process of sexual development.

To investigate the attitudes and practices of parents in Qaemshahr, north of Iran, regarding sexual health education, 600 parents who had children 0–12 yr old, completed a survey as Children Sexuality Management Questionnaire, in 2015.

The study was approved by Ethics Committee of Shahroud University of Medical Sciences with the ethical code IR.SHMU.REC.2015.48. Informed consent was taken from all participants.

In this study 77.6% had a positive attitude towards children’s sexual education. Although a majority of 78.2% believed that parents are children’s first educators with respect to sexuality, most of them (82.2%) had not discussed any of a range of sexual health education topics with their children, and 88.7% of parents did not know how to react to their children’s sexual behaviors.

Parents have an important role in sexual education. However, they usually have some barriers to do this education and often are not successful to educate their children in proper time and in both genders (3–6). Only one-fourth of parents frequently discussed sexual issues with their children and many parents had difficulty in discussing important topics such as condom use (7). There is not any formal and informal education about children sexuality in Iran, lead to parents’ difficulty to manage sexual behavior of their children (1). In many countries, due to the different reasons, schools have a little role in sexual education and therefore families have the main responsibility for this matter (8). Family-based sex education is one of the topics that can empower parents to be effective in response to the sexual behavior of their children. Family-based sex education methods are necessary for teaching children (4). This training leads to an improvement in knowledge, attitudes, and practices of parents’ sexual issues and affects the quality of sexuality education for children (5). Therefore, in order to response to children’s sexual behaviors; an educational package should be designed to empower the parents, who are the first sexual educators of children.
